# Semi-Supervised Medical Image Segmentation Based on Deep Consistent Collaborative Learning

**DOI:** 10.3390/jimaging10050118

**Published:** 2024-05-14

**Authors:** Xin Zhao, Wenqi Wang

**Affiliations:** College of Information Engineering, Dalian University, Dalian 116622, China; wwenqi238@gmail.com

**Keywords:** semi-supervised learning, medical image segmentation, consistent regularization, co-training

## Abstract

In the realm of medical image analysis, the cost associated with acquiring accurately labeled data is prohibitively high. To address the issue of label scarcity, semi-supervised learning methods are employed, utilizing unlabeled data alongside a limited set of labeled data. This paper presents a novel semi-supervised medical segmentation framework, DCCLNet (deep consistency collaborative learning UNet), grounded in deep consistent co-learning. The framework synergistically integrates consistency learning from feature and input perturbations, coupled with collaborative training between CNN (convolutional neural networks) and ViT (vision transformer), to capitalize on the learning advantages offered by these two distinct paradigms. Feature perturbation involves the application of auxiliary decoders with varied feature disturbances to the main CNN backbone, enhancing the robustness of the CNN backbone through consistency constraints generated by the auxiliary and main decoders. Input perturbation employs an MT (mean teacher) architecture wherein the main network serves as the student model guided by a teacher model subjected to input perturbations. Collaborative training aims to improve the accuracy of the main networks by encouraging mutual learning between the CNN and ViT. Experiments conducted on publicly available datasets for ACDC (automated cardiac diagnosis challenge) and Prostate datasets yielded Dice coefficients of 0.890 and 0.812, respectively. Additionally, comprehensive ablation studies were performed to demonstrate the effectiveness of each methodological contribution in this study.

## 1. Introduction

Image segmentation algorithms based on supervised learning have demonstrated outstanding performance in a wide range of tasks [1,2,3], benefiting from their reliance on large amounts of high-quality, pixel-level annotated data, enabling models to learn precise segmentation decisions. However, the substantial demand for annotated data is not always feasible in practical applications, especially in the field of medical image segmentation. Due to the complexity of medical images and the high requirement for annotation accuracy, annotation tasks must be manually performed by experienced experts, which is not only time-consuming and labor-intensive but also costly. Therefore, in the field of medical image segmentation, the reality often involves only a small amount of annotated data available for use, while there is a relatively large amount of unlabeled data. Many semi-supervised segmentation algorithms have limited learning capabilities for the features of unlabeled data under conditions of a small amount of annotated data, resulting in low image segmentation accuracy. In this situation, effectively utilizing limited annotated data for training becomes a challenge in medical image segmentation.

An effective solution is to adopt semi-supervised learning methods. Currently, semi-supervised methods proposed based on deep learning mainly include consistency learning [4], co-training [5], self-training [6], adversarial learning [7], entropy minimization [8], and other methods. Among these methods, consistency regularization and co-training are widely used.

The consistency learning method is based on the assumption that the output should not change significantly when the input data are slightly perturbed. This method encourages the model to produce similar outputs for similar inputs, thereby enhancing the model’s robustness. According to the research in [9], the co-training framework is established on the premise that each data sample has two independent views, and each view can make predictions independently, with one view being redundant to the other. Simultaneously, this framework encourages the model to make consistent predictions for these two views. Initially, a segmentation model is separately trained for each view on the labeled data, and then the predictions of these two models for the unlabeled data are gradually introduced into the training set for continued training. The difference between co-training and consistency learning lies in that co-training utilizes pseudo-labels generated from unlabeled data to guide the training of models other than itself, while consistency learning forces the model’s output to remain consistent under different types of perturbations.

To address the challenges of low segmentation accuracy and inadequate generalization ability in the medical domain under conditions of scarce labels, this chapter proposes a semi-supervised medical image segmentation model named DCCLNet (deep consistency collaborative learning UNet) based on deep consistent co-training learning. This model combines the performance of consistency learning with both feature and input perturbations, and effectively leverages the advantages of learning different paradigms in co-training. The consistency learning strategy is primarily manifested by adding different auxiliary decoders on top of the backbone network for auxiliary learning, while employing a teacher–student architecture composed of a teacher model and the backbone network. The teacher model guides the learning of the backbone network, with the backbone network acting as the student model. Meanwhile, the co-training framework comprises two different network architectures, CNN (convolutional neural networks) and ViT (vision transformer), trained simultaneously and mutually learning from each other. Overall, the main contributions of this chapter are as follows:

(1) Proposed a semi-supervised segmentation model named DCCLNet based on deep consistent co-training learning. Inspired by the CCT (cross-consistency training) semi-supervised method [10], this model adds different feature perturbations to the output of the backbone network’s CNN encoder, which are then inputted into auxiliary decoders. This encourages consistency between the outputs of the main decoder and the auxiliary decoder, thereby enhancing the robustness of the backbone network CNN.
(2) Added a teacher model to form an MT (mean teacher) architecture [11] with the backbone network. Data with input perturbations are inputted into the teacher model, and a consistency constraint is imposed between the predictions of the teacher model and the backbone network to guide the training of the backbone network further, thereby improving the robustness and accuracy of the backbone network CNN. Moreover, the parameters of the teacher model are obtained from the backbone network, effectively reducing computational complexity.
(3) Utilized the backbone network CNN and ViT to form a co-training architecture, where CNN can better capture local features, and ViT can better capture long-range dependencies. By simultaneously training from the perspectives of two different network architectures and learning pseudo-labels generated from each other’s predictions, the accuracy of the backbone network CNN can be improved.
(4) Conducted experiments on the ACDC dataset [12] and Prostate dataset [13] under different percentages of labeled data. Experimental results demonstrate that DCCLNet outperforms other semi-supervised segmentation methods on the ACDC dataset and Prostate dataset in terms of segmentation effectiveness.

The subsequent structure of the paper is as follows: Section 2 reviews the research on semi-supervised medical image segmentation methods and prior studies related to the model we propose. Section 3 provides an in-depth introduction to the various modules of the proposed model. Section 4 and Section 5 delve into a thorough discussion of the experimental results. Finally, Section 6 concludes with considerations for future directions of this research.

## 2. Related Works

### 2.1. Semi-Supervised Medical Image Segmentation

Recently, the importance of semi-supervised learning in the field of medical image analysis has been increasing, and the use of semi-supervised semantic segmentation methods has become increasingly widespread, which is an excellent method for reducing the cost of image annotation. For example, Bai et al. [14] proposed a semi-supervised method for cardiac MR image segmentation. The training regimen alternates between labeled and unlabeled datasets, with pseudo-labels generated for the unlabeled data to facilitate segmentation of the originally labeled data. Xu et al. [15] proposed a semi-supervised segmentation method tailored for transrectal ultrasound (TRUS) images. The method leverages shadow consistency, which encompasses both shadow enhancement and shadow loss, to replicate the effects of low image quality and shadow artifacts commonly found in medical imaging. Shadow enhancement is achieved by superimposing synthetically generated shadow artifacts onto the input images, whereas shadow loss induces neural node degradation based on prior understanding of shadow artifacts, directly impacting the feature map. Zhu et al. [16] introduced an asymmetric multi-modal deep co-training framework for semi-supervised medical image segmentation, which includes two segmentation networks and two image translation networks designed for cross-modality image translation. Labeled data from one modality are utilized to train the segmentation network of the other modality, with consistency constraints applied to minimize the discrepancy between the two segmentation networks. Zhao et al. [17] presented a semi-supervised segmentation method featuring an information self-integration architecture for 3D whole-brain segmentation. The method employs various transformation strategies for unlabeled data, with the averaged prediction outcomes serving as pseudo-labels for the unlabeled data. Wu et al. [18] introduced a semi-supervised polyp segmentation model that leverages two segmentation networks to learn from each other. The approach utilizes a discriminator to generate confidence maps and incorporates an auxiliary discriminator to aid the primary discriminator, which may face performance limitations due to the scarcity of labeled data. Hou et al. [19] proposed a semi-supervised medical image segmentation method that employs a Leaking GAN (generative adversarial network) to contaminate the discriminator by leaking information from the generator, thereby promoting better learning of the discriminator. Wu et al. [20] introduced a semi-supervised segmentation method that augments the inter-class distance within the feature space by means of feature gradient map regularization. Additionally, the method employs class consistency constraints to alleviate the impact of noise interference on pseudo-labels. Wu et al. [21] presented a semi-supervised nuclei segmentation method that enhances the intra-class compactness and inter-class separability of features by aligning the feature maps of a teacher model with those of a student network, sampling from cross-image patches and pixel-level images. Miao et al. [22] proposed a semi-supervised medical image segmentation method with self-correcting collaborative training, which employs a self-correcting module to enhance the accuracy of model predictions on unlabeled data and further improves the model’s learning capability using pixel-wise contrastive learning loss. Shen et al. [23] proposed a UCMT (uncertainty-guided collaborative mean teacher) model for semi-supervised medical image segmentation, where sub-networks learn from unlabeled data through collaborative training, and uncertainty estimation strategies are applied to predictions of the teacher network, allowing sub-networks to learn from regions of high confidence.

### 2.2. Consistent Learning

The consistency learning method imposes consistency on the predictions of input images under different perturbations, where perturbations should not alter the network’s predictions. Various perturbations are employed in consistency learning methods, which can roughly be categorized into input perturbations and feature map perturbations.

Input perturbation refers to applying certain perturbations to the input samples, such as Gaussian noise, random rotation, flipping, and contrast changes, encouraging segmentation networks to maintain consistency in predictions for unlabeled data under different perturbations. The Π-model proposed by Sajjadi et al. [24] and the MT (Mean Teacher) method proposed by Tarvianen et al. are similar approaches. The former applies two versions of data perturbation to samples and adds a consistency constraint between their predictions, while the latter improves upon the former by obtaining the weights of the student model through training, and the weights of the teacher model are continually updated through exponential moving average (EMA) calculation based on the weights of the student model, imposing consistency constraints between the predictions of the teacher and student. The ICT (Interpolation Consistency Training) [25] method introduces a training approach based on interpolation consistency, which interpolates unlabeled data according to the idea of mix-up [26]. Mix-up involves applying a simple linear transformation to input data. ICT calculates consistency loss between predictions at interpolated pixel points of unlabeled data and the predictions of those points themselves, training the network through consistency constraints between them. Huang et al. [27] proposed a CC-Net based on complementary consistency training for semi-supervised left atrial image segmentation. The method utilizes two auxiliary models and introduces model-level perturbations, enhancing the learning capability of the main model through the imposition of consistency constraints between the auxiliary and main models. Although CC-Net facilitates ample assistance for the backbone network’s learning through two auxiliary models with model perturbations, employing identical encoder and decoder structures may potentially diminish the efficacy of co-training.

Feature perturbation involves perturbations added to the feature maps after convolution of input samples. The CCT (Cross Consistency Training) method proposes a cross-consistency approach, where unlabeled data are input into a shared encoder, and different feature perturbations are added to the output of the shared encoder, which are then inputted into auxiliary decoders. The network model is trained by imposing consistency loss between the predictions of the auxiliary decoder and the main decoder. The RD (Regularized Dropout) [28] method introduces a simple consistency training strategy called R-Drop, which enforces consistency between the outputs of different sub-models through dropout. Li et al. [29] introduced a semi-supervised segmentation approach for COVID-19 based on an uncertainty-guided dual consistency learning network (UDC-Net). UDC-Net incorporated seven types of feature perturbations for seven additional auxiliary decoders, necessitating that these seven predictions remain consistent with the main decoder. The feature-level perturbations included feature noise, feature dropout, object masking, context masking, guidance loss, intermediate boosting, and random loss. This method is very comprehensive in its use of feature perturbations but overlooks that input perturbations can also mislead the encoder’s feature learning during the encoding process.

### 2.3. Co-Training

The co-training method involves constructing two or more network models capable of representing independent perspectives. Typically, different network architectures and specialized training methods are used to build co-training models. The CPS (Cross Consistency Training) [30] method proposes a cross-pseudo-supervised training approach, employing two networks that are structurally consistent but differ in initialization state. It introduces consistency constraints to encourage these two networks to produce the same output for the same input sample. CPS generates labels from the predictions of one network and uses them as pseudo-labels for the predictions of the other network, and vice versa, allowing these two networks to learn from each other’s perspective. The CTCT (Cross Teaching between CNN and Transformer) [31] method further proposes mutual teaching between two different networks utilizing CNN and transformer architectures, enabling them to observe data from two completely different perspectives and learn from each other, thus compensating for the limitations of learning with a single CNN network architecture. The DCT (Deep Co-Training) [32] method introduces a deep co-learning approach, using multiple deep neural networks to observe data from multiple perspectives and impose constraints by leveraging differences generated by adversarial samples. Wang et al. [33] proposed a semi-supervised medical image segmentation framework, consisting of a feature learning module composed of CNN and ViT, and a feature-guidance module composed of a ViT teacher model. The feature learning module combines the advantages of CNN and ViT through dual-view co-training. The feature guidance module averages network weights using an MT architecture and considers the output of the teacher model as the final prediction. The framework employed two different network architectures, and to further guide the ViT, the framework did not consider the potential negative effects of feature perturbations. Huang et al. [34] developed a semi-supervised dual cooperative network (SD-Net) for liver CT segmentation, which adopts two cooperatively trained models. They used adaptive mask refinement to refine the differences between predictions for labeled data and utilized a dynamic pseudo-label-generation strategy for unlabeled data to select better predictions as pseudo-labels. This method utilizes two networks to collaborate and imposes constraints on the uncertain regions predicted by both networks. However, both network architectures are CNN-based, lacking the advantage of ViT in capturing long-range dependencies.

## 3. Method

### 3.1. The Overall Structure of the Model

**Figure 1:**
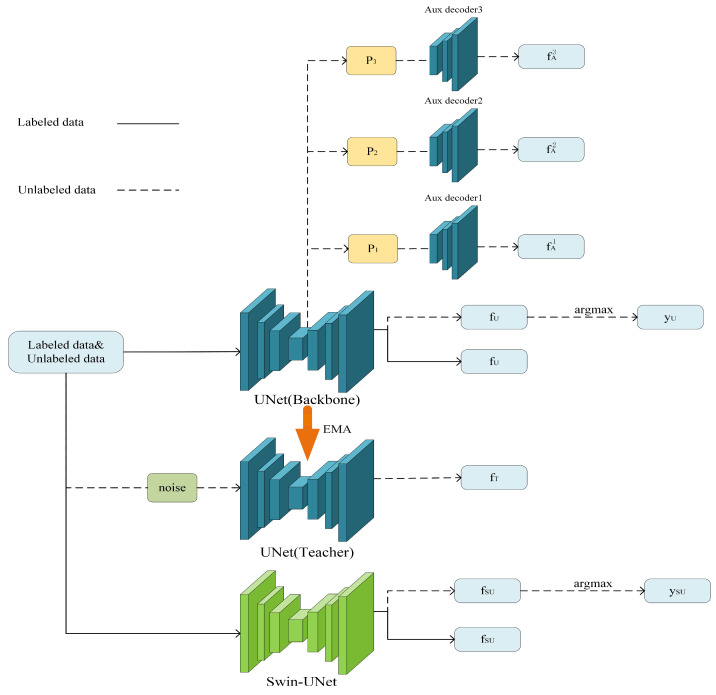
Overall framework DCCLNet. where P1, P2, P3 are three different characteristic perturbations.

The overall model architecture of DCCLNet is shown in Figure 1. The model is primarily based on the backbone network UNet [35], with three auxiliary decoders added on the basis of the main decoder of the UNet backbone network, each applying different feature perturbations. Additionally, a teacher model with input perturbation is added, which shares the same UNet network structure as the UNet backbone network, along with Swin-UNet [36].

In semi-supervised segmentation, images are typically divided into two parts: labeled images and unlabeled images. A dataset with N labeled images is represented as $\left(x_{l},y_{l}\right)\in L$, while a dataset with M unlabeled images is represented as $\left(x_{u}\right)\in U$.

The labeled data are only received by the backbone networks UNet and Swin-UNet, while the unlabeled data are received by all networks in the framework. For the labeled data, the predictions generated by DCCLNet are compared only to the ground truth labels and continually learned from them. For the unlabeled data, DCCLNet guides the backbone network UNet with different levels of perturbation by adding auxiliary decoders with feature perturbation and a teacher model with input perturbation. Consistency constraints are imposed by the differences between their predictions and those of the backbone network UNet. Additionally, DCCLNet employs a collaborative training approach using two different network architectures, UNet and Swin-UNet, to learn from each other.

### 3.2. Auxiliary Decoder Assist

Inspired by the CCT semi-supervised method, three different types of feature perturbations are added to the output of the backbone network encoder, which are then respectively inputted into the auxiliary decoders. Based on the principle of consistency learning, perturbing the feature maps of the decoder input should not change the output predictions. By imposing consistency between the predictions of the backbone network and the auxiliary decoders, the generalization ability of the backbone network UNet is enhanced.

**Figure 2:**
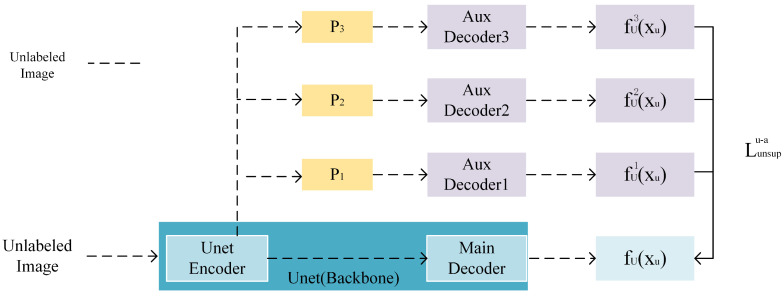
Auxiliary decoder and backbone network UNet.

As shown in Figure 2, the decoder structure of the auxiliary decoder is the same as that of the backbone network UNet, and they share an encoder. Here, P_1_, P_2_, and P_3_ represent three different types of feature perturbations. Unlabeled data are input into the shared UNet encoder, whereas the main decoder receives the direct output of the encoder, and the auxiliary decoders respectively add these three types of feature perturbations to the output feature maps of the encoder. This enables the auxiliary decoders to assist the backbone network in learning from feature perturbations.

#### 3.2.1. Characteristic Perturbation

Feature Noise: Uniformly sample a noise tensor N~U(-3,3) with the same size as the feature map *h*, then multiply this noise tensor with *h*, injecting the noise into *h*, resulting in the perturbed result $h\prime =\left(h\circ N\right)+h$. The injected noise is proportional to each activation.

Feature Dropout: Uniformly sample a threshold value *γ*~U(0.6,0.9), perform channel-wise summation and normalization on the feature map *h* to obtain $\overset{\wedge }{h}$, generate a mask $F_{drop}=\left\{h<\gamma \right\}$, and use this mask to add perturbation to *h*, resulting in $h^{\prime }=F_{drop}\circ \overset{\wedge }{h}$, effectively masking the most active regions of *h* by 10% to 40%.

Dropout: Use Dropout [37] for feature map *h* generation as random perturbations.

#### 3.2.2. Characteristic Perturbation Loss

For the unlabeled dataset $\left(x_{u}\right)\in U$, let the output of the backbone network be represented as $f_{U}\left(x_{u}\right)$, and the output of the auxiliary decoder Aux be represented as $f_{A}^{i}\left(x_{u}\right),i=1,2,3$. The difference between the predictions of the backbone network UNet and the auxiliary decoder Aux is constrained by the unsupervised loss $L_{un\sup}^{u-a}$ constructed by mean squared error (MSE):

(1) $$L_{un\sup}^{u-a}=\frac{1}{3}\times \sum_{i=1}^{3}MSE\left(f_{U}\left(x_{u}\right),f_{A}^{i}\left(x_{u}\right)\right)$$

### 3.3. Teacher Model Guidance

Addition of the teacher model forms the MT architecture with the backbone network UNet, where the backbone network acts as the student model. Perturbations are added to the inputs of the teacher model based on the principle that perturbations should not alter the model’s output. Consistency constraints are imposed between the predictions of the backbone network and the teacher model, further enhancing the robustness of the backbone network.

**Figure 3:**
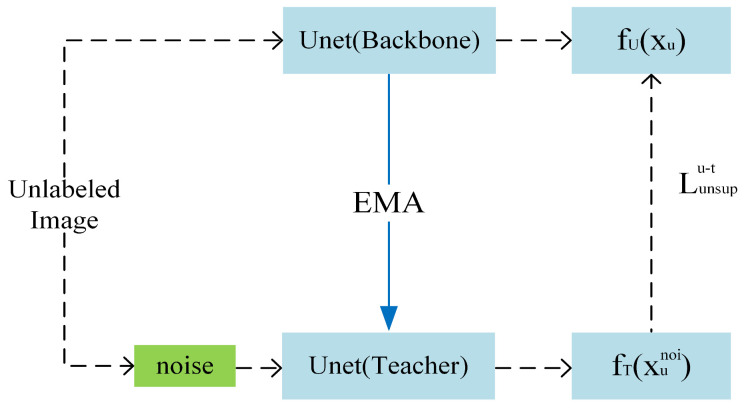
The framework of MT (mean teacher) (EMA: exponential moving average).

As shown in Figure 3, both the teacher model and the backbone network are UNet networks. The parameters of the teacher model are directly obtained from the backbone network UNet through exponential moving average (EMA), effectively reducing the computational cost of the model. Unlabeled data are directly input into the backbone network UNet, while after adding noise, it is input into the teacher model. This enables the teacher model to guide the backbone network in learning from input perturbations.

#### 3.3.1. Teacher Model Parameter Update

The study in [11] indicates that averaging model weights during training often results in models that are more accurate than those directly using the final weights. Therefore, the EMA method is utilized to update the parameters of the teacher model. Let *θ_s_* denote the parameters of the student model, and the update calculation for the parameters *θ_t_* of the teacher model is as follows:

(2) $$\theta_{t}=\lambda \theta_{t-1}+\left(1-\lambda \right)\theta_{s}$$

where *λ* is the decay coefficient used to control the updating rate of EMA, and *t* denotes the updating round.

#### 3.3.2. Input Disturbance Loss

For the unlabeled dataset $\left(x_{u}\right)\in U$, let the data with added input perturbation be denoted as $x_{u}^{noi}$, and the output of the teacher model for the unlabeled data is represented as $f_{T}\left(x_{u}^{noi}\right)$. The discrepancy between the predictions of the main network and the teacher model is computed through the unsupervised loss $L_{un\sup}^{u-t}$, which is based on the mean square error.

(3) $$L_{un\sup}^{u-t}=MSE\left(f_{U}\left(x_{u}\right),f_{T}\left(x_{u}^{noi}\right)\right)$$

### 3.4. CNN and ViT Collaborative Training

#### 3.4.1. Collaborative Training Process

In reference [31], a semi-supervised segmentation method was proposed, which involves the cross-teaching of UNet and Swin-UNet, yielding impressive segmentation results. Consequently, this paper utilizes a collaborative training framework composed of the CNN-based UNet and the Transformer-based Swin-UNet, which are trained concurrently. Through the observation of data by two different network architectures and mutual learning during the training process, the advantages of each are leveraged to compensate for shortcomings, effectively enhancing the segmentation accuracy of the main network UNet.

**Figure 4:**
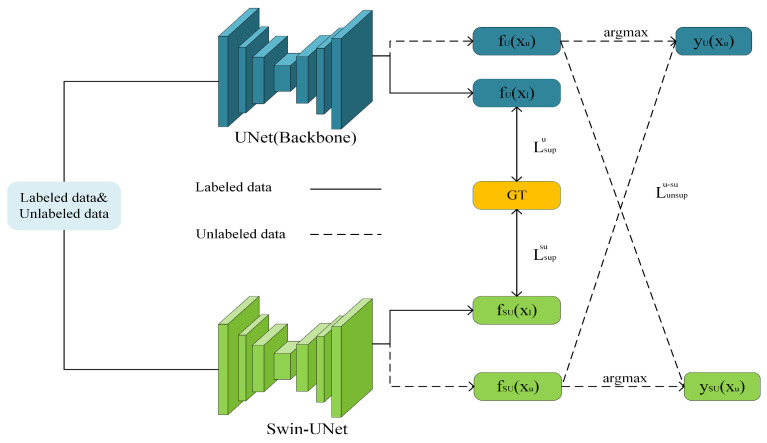
UNet and Swin-UNet collaborative training framework.

As illustrated in Figure 4, when inputting labeled data, the predictions of the main network UNet and Swin-UNet are constrained by the ground truth labels. When inputting unlabeled data, the labels generated by Swin-UNet are utilized to guide the predictions of UNet. Simultaneously, the labels generated by UNet are used to guide the predictions of Swin-UNet. Thus, the objective is achieved where UNet and Swin-UNet mutually learn and progress together.

#### 3.4.2. Co-Training Loss

For the labeled dataset $\left(x_{l},y_{l}\right)\in L$, let the outputs of the main network UNet be denoted as $f_{U}\left(x_{l}\right)$ and the outputs of Swin-UNet be denoted as $f_{SU}\left(x_{l}\right)$. The differences between them and the ground truth labels are calculated using the supervised losses constructed by the cross-entropy loss function (CE) and the Dice loss function, denoted as $L_{\sup}^{u}$ and $L_{\sup}^{su}$, respectively:

(4) $$L_{\sup}^{u}=\frac{1}{2}\left(CE\left(f_{U}\left(x_{l}\right),y_{l}\right)+Dice\left(f_{U}\left(x_{l}\right),y_{l}\right)\right)$$

(5) $$L_{\sup}^{su}=\frac{1}{2}\left(CE\left(f_{SU}\left(x_{l}\right),y_{l}\right)+Dice\left(f_{SU}\left(x_{l}\right),y_{l}\right)\right)$$

For the unlabeled dataset $\left(x_{u}\right)\in U$, let the predictions of the main network UNet be denoted as $f_{U}\left(x_{u}\right)$ with generated labels $y_{U}\left(x_{u}\right)=\arg\max\left(f_{U}\left(x_{u}\right)\right)$, and the predictions of Swin-UNet be denoted as $f_{SU}\left(x_{u}\right)$ with generated labels $y_{SU}\left(x_{u}\right)=\arg\max\left(f_{SU}\left(x_{u}\right)\right)$. On one hand, the predictions $f_{U}\left(x_{u}\right)$ of the main network and the labels $y_{SU}\left(x_{u}\right)$ generated by Swin-UNet are used to compute the loss. On the other hand, the predictions $f_{SU}\left(x_{u}\right)$ of Swin-UNet and the labels $y_{U}\left(x_{u}\right)$ generated by the main network are used to compute the loss. The differences between them are calculated by the unsupervised loss $L_{un\sup}^{u-su}$:

(6) $$L_{un\sup}^{u-su}=Dice\left(f_{U}\left(x_{u}\right),y_{SU}\left(x_{u}\right)\right)+Dice\left(f_{SU}\left(x_{u}\right),y_{U}\left(x_{u}\right)\right)$$

### 3.5. Overall Loss Function

The loss of the semi-supervised framework consists of the supervised loss $L_{\sup}$ and the unsupervised loss $L_{un\sup}$. In the overall framework of DCCLNet proposed in this paper, the training of the auxiliary decoder, the main network UNet, the teacher model, and Swin-UNet are conducted simultaneously. Therefore, the overall loss function $L_{total}$ of DCCLNet is composed of all the aforementioned loss functions:

(7) $$L_{un\sup}^{u-su}=Dice\left(f_{U}\left(x_{u}\right),y_{SU}\left(x_{u}\right)\right)+Dice\left(f_{SU}\left(x_{u}\right),y_{U}\left(x_{u}\right)\right)$$

In this equation, λ represents the weight of the unsupervised loss. Different weights are used for consistency learning and collaborative training in this paper, where $\lambda_{1}$ is the weight factor for the consistency loss of the main UNet network, $\lambda_{1}=0.01\times e^{-5\times {\left(1-t/t_{\max}\right)}^{2}}$; $\lambda_{2}$ is the weight factor for the collaborative training loss of the main UNet network, $\lambda_{2}=u\times \lambda_{1}$; and *u* is a scaling factor. *t* denotes the current iteration round, updated every 150 iterations and gradually increased during training [38], allowing the model to focus on labeled images during initialization and gradually shift the focus to unlabeled images. The overall loss $L_{total}$ is the final training objective of this framework.

## 4. Experiments

### 4.1. Data Preparation

ACDC dataset: The semi-supervised segmentation method proposed in this paper is evaluated on the MICCAI 2017 ACDC (Automated Cardiac Diagnosis Challenge) dataset to demonstrate its effectiveness and compare it with other baseline methods. The ACDC dataset consists of 200 short-axis cardiac cine MRI scans from 100 patients, each containing four segmentation classes: left ventricle, right ventricle, left ventricular myocardium and background. Among the 100 patients, 70 were selected as training samples, 10 as validation samples, and 20 as testing samples.

PROMISE12 dataset: Similar experiments to those on the ACDC dataset are conducted on the PROMISE12 (Prostate MR Image Segmentation Challenge) dataset. The PROMISE12 dataset comprises 50 MRI scans from patients, with 35 patients used for training, 5 for validation, and 10 for testing, each containing two segmentation classes: prostate and background.

### 4.2. Experimental Setup

To ensure fair comparison among all methods, all comparative methods and ablation experiments in this study were conducted under the same conditions. The experiments were performed using an NVIDIA GeForce RTX 2080 Ti GPU (NVIDIA, Dalian, China) with retraining in PyTorch 3.9. The datasets were normalized and resized to 224 × 224, and data augmentation techniques such as random rotation and flipping were applied. An SGD (stochastic gradient descent) optimizer was used during training with an initial learning rate of 0.01, and the Poly learning rate policy was employed to update the learning rate continuously. The training process consisted of 30,000 iterations, with a batch size of 16, including 8 labeled data and 8 unlabeled data per batch. The scaling factor *u* of DCCLNet was set to 15. The optimal weights of the backbone network in DCCLNet were used to generate the final predictions.

### 4.3. Evaluation Index

This paper evaluates the performance of the proposed model using three standard evaluation metrics: Dice similarity coefficient (DSC), 95% Hausdorff distance (HD_95_), and average symmetric surface distance (ASD). The formulas for calculation are as follows:

(8) $$DSC=\frac{2TP}{FN+2TP+FP}$$

DSC mainly measures the similarity between two segmentation maps, where TP is true positive, FP is false positive, TN is true negative, and FN is false negative. DSC ranges from [0, 1], with values closer to 1 indicating better segmentation results.

(9) $$HD_{95}=\underset{t2}{\max}\left(\underset{t1}{\max}\left(\sqrt{T^{2}-P^{2}}\right)\right)\times 0.95$$

HD represents the maximum distance measure between the surface point sets of the predicted result and the ground truth label. Since the Hausdorff distance metric is affected by noisy points, the 95th percentile of the Hausdorff distance is used to measure the segmentation result, where *T* is the boundary of the label region and *P* is the boundary of the segmented region predicted by the model.

(10) $$ASD=\frac{1}{S\left(A\right)+S\left(B\right)}\left(\sum_{s_{A}\in S\left(A\right)}d\left(s_{A},S\left(B\right)\right)+\sum_{s_{B}\in S\left(B\right)}d\left(s_{B},S\left(A\right)\right)\right)$$

ASD represents the average distance between the surface point sets of the predicted result and the ground truth label, where *S*(*A*) represents the surface voxels in set *A*, and *d*(*v*, *S*(*A*)) represents the shortest distance from any voxel to *S*(*A*); HD_95_ and ASD measure the segmentation model’s ability to predict edges, with smaller values indicating that the segmented edges are closer to the edges of the ground truth label.

### 4.4. Comparative Experimental

#### 4.4.1. ACDC Dataset Comparative Experimental Analysis

As shown in Table 1, this paper utilized two different percentages of labeled datasets from the ACDC training set, namely 5% and 10%, and conducted experiments with other semi-supervised segmentation methods for these two scenarios. The proposed framework DCCLNet achieved better performance on most metrics.

When using 5% labeled samples, consistency-based methods like CCT, MT, and ICT performed poorly on all three metrics. This is because these three consistency methods only employ a single network architecture. CCT solely assists the main network in learning from unlabeled data by adding auxiliary decoders with feature perturbations; MT also only utilizes a teacher model with the same architecture to guide the student model, where the teacher model’s input is perturbed; ICT uses interpolation to train on perturbed data. UAMT, building upon MT, incorporates uncertainty estimation to select more reliable pseudo-labels for guiding the student model. However, when the labeled data volume is 5%, the available labeled data for training after selection is too scarce, resulting in inferior performance compared to MT. URPC generates multi-scale predictions through a pyramid framework, to which consistency loss and uncertainty correction are applied. EM improves pseudo-label quality by reducing the entropy of pseudo-labels, but due to the scarcity of labels and a single network, the effect is poor. DAN also performs poorly as adversarial training is not applicable in this scenario. CPS supervises the pseudo-labels of each other through two networks with the same architecture, which enhances label quality with acceptable performance. DCT increases adversarial sample loss by adding noise to generate adversarial samples, but the accuracy of labels corresponding to adversarial samples is insufficient due to the use of a single network for label generation. RD trains the network using only a regularization loss, resulting in the worst performance. CTCT exhibits excellent performance, employing cross-teaching between CNN and Transformer, effectively improving segmentation accuracy by mutual learning from two different nework architectures. Compared to CTCT, the proposed method DCCLNet shows signifcant improvements in all metrics. Specifically, DSC, HD_95_, and ASD increased by 7.8%, 1.5 mm, and −1.0 mm, respectively, indicating that DCCLNet can maintain good performance with fewer labeled data.

**Figure 5:**
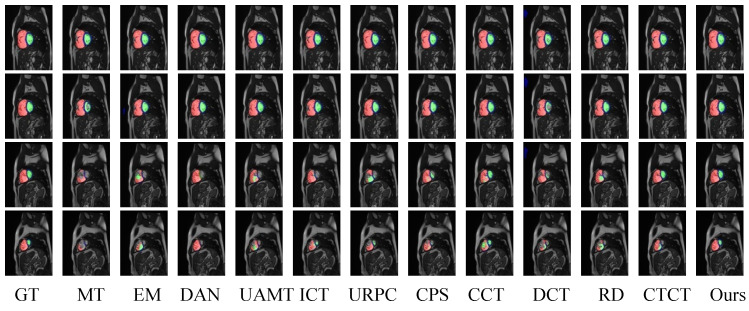
Visualization results of different methods on the ACDC test set. In the image, red denotes the right ventricle, blue denotes the myocardium, and green denotes the left ventricle.

When using 10% of the samples, CCT and CPS performed relatively well, indicating that the potential of cross-pseudo-supervision and feature perturbation methods can be better utilized with more labeled data. CTCT still outperformed other semi-supervised segmentation methods without ViT. Compared to CTCT, DCCLNet showed improvements in DSC, HD_95_, and ASD by 2.4%, −2.3 mm, and −0.1 mm, respectively, indicating that DCCLNet learns more effectively from unlabeled data, and the feature perturbation and input perturbation added in the framework also achieved certain effects. In Figure 5, the final results of all methods trained and tested with 10% labeled samples are visualized. It can be seen from Figure 5 that DCCLNet’s segmentation performance has notable improvements in both boundary and overall accuracy compared to other methods.

#### 4.4.2. Prostate Dataset Comparative Experimental Analysis

As shown in Table 2, this paper utilized two different percentages of labeled datasets from the Prostate training set, namely 10% and 20%. On this dataset, DCCLNet achieved the best results in terms of DSC, HD_95_, and ASD.

**Figure 6:**
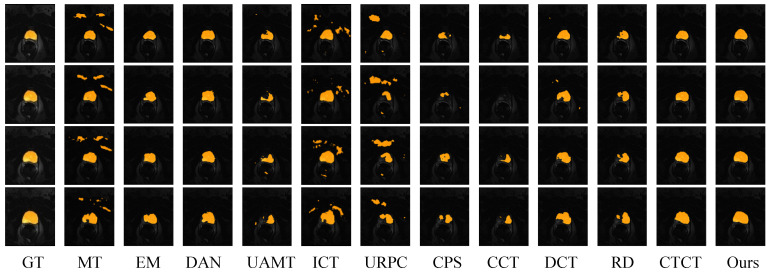
Visualized results of different methods on the PROMISE12 test set. In the image, yellow represents the prostate, while the rest constitutes the background.

When using 10% labeled samples, it is evident that the segmentation performance of the majority of semi-supervised segmentation methods is poor, even when segmenting only the prostate organ. This indicates that when labeled data is extremely scarce, the segmentation results from single-network architectures are subpar. URPC and CPS methods performed extremely poorly, with DSC values even below 0.35. The segmentation results of the CTCT method were relatively good, suggesting that even with very few labeled data, decent results can be obtained through the use of CNN and ViT, two drastically different learning paradigms. Compared to CTCT, DCCLNet showed improvements in all metrics, with DSC, HD_95_, and ASD values increasing by 2.9%, −7.6 mm, and −2.0 mm, respectively. This demonstrates that the deep integration of consistency regularization methods and CNN-ViT collaborative training methods can enhance model performance. Figure 6 visualizes the segmentation results of the DCCLNet method and other semi-supervised methods on the Prostate dataset.

### 4.5. Ablation Experiment

The ablation study results of DCCLNet on the ACDC dataset using 10% labeled data are presented in Table 3, while those on the Prostate dataset with 20% labeled data are shown in Table 4. As DCCLNet employs the UNet network as its backbone, the experimental results of UNet are used as the baseline to assess the effectiveness of the proposed improvements in this study.

For the results of the ablation experiments on these two datasets, the study first investigated the segmentation performance when only a single component was added to UNet, namely UNet + Aux, UNet + Tea, and UNet + ViT. All three architectures exhibited better experimental results compared to the baseline, indicating that adding feature perturbation or input perturbation, as well as using different network architectures for co-training, can enhance the learning of the backbone network for unlabeled data. Subsequently, experiments were conducted by combining any two components, all of which yielded excellent results, indicating that both the combination of perturbations and the combination of perturbations with co-training can further improve the learning ability of the backbone network. However, it was also observed that the improvement achieved by the co-training architecture of CNN with ViT was significant, outperforming the direct addition of perturbations. This is because medical image perturbations lead to significant data variations, and a single UNet network architecture is insufficient to learn enough knowledge for correction. Finally, applying all components to the backbone network yielded better results than all previous scenarios.

The experimental data in Table 3 demonstrate that for the DSC evaluation metric, DCCLNet outperformed the baseline by 11.2 percentage points, and for the HD_95_ and ASD evaluation metrics, it improved by −7.8 mm and −1.4 mm, respectively. Compared to the framework with only perturbations added (UNet + Aux + Tea), DCCLNet improved by 2.4 percentage points in DSC score and by −2.3 mm and −0.1 mm in HD_95_ and ASD distances, respectively. Compared to the framework with only co-training architecture used (UNet + ViT), DCCLNet improved by 6.4 percentage points in DSC score and by −7.9 mm and −1.1 mm in HD_95_ and ASD distances, respectively.

The experimental data in Table 4 indicate that for the DSC evaluation metric, DCCLNet outperforms the baseline by 24.9 percentage points, while for the HD_95_ and ASD evaluation metrics, it improves by −76 mm and −18.2 mm, respectively. Compared to the framework that only adds perturbations (UNet + Aux + Tea), DCCLNet achieves an increase of 11.7 percentage points in DSC score, and improvements of −13.9 mm and −3.7 mm in HD_95_ and ASD distances, respectively. Compared to the framework using only collaborative training (UNet + ViT), DCCLNet shows an increase of 2.9 percentage points in DSC score, with improvements of −7.6 mm and −2.0 mm in HD_95_ and ASD distances, respectively.

## 5. Discussion

In response to the challenges and issues in current semi-supervised medical image segmentation tasks, this paper proposes a semi-supervised medical image segmentation model called DCCLNet based on deep consistent co-training learning. The model combines the performance of consistency learning with feature perturbation and input perturbation, effectively leveraging the advantages of learning from different paradigms in co-training. When applied to various medical datasets, the segmentation results are outstanding even with limited annotated data. Although this study has made some progress, there are also shortcomings.

Firstly, although the semi-supervised learning method proposed in this study can fully learn the feature information of unlabeled data and enhance network generalization ability, there is still room for improvement in segmentation performance compared to supervised learning algorithms. Therefore, future research will focus on further developing semi-supervised segmentation algorithms to make their performance approach that of fully supervised methods.

Secondly, this study introduced feature perturbation and input perturbation from the perspective of perturbations to help the backbone network learn knowledge from unlabeled data and enhance its robustness. Additionally, from the standpoint of co-training, the combination of CNN and ViT architectures mutually learned from each other, effectively improving the accuracy of the backbone network. Although the improved semi-supervised segmentation model further enhances the accuracy of medical image segmentation, it also requires more computational resources. Future work could focus on more efficiently combining CNN and ViT with fewer computational requirements to improve model performance and reduce parameter count.

Moreover, this study involves setting numerous hyperparameters, including learning rates and weights of unsupervised losses, which need to be explored through continuous experimentation. Hence, future research directions could explore more automated hyperparameter optimization strategies.

## 6. Conclusions

Supervised image segmentation algorithms have demonstrated outstanding performance in a wide range of tasks, owing to their reliance on large amounts of high-quality, pixel-level annotated data, which allows models to learn precise segmentation decisions. However, this heavy reliance on annotated data is not always feasible in practical applications, especially in the field of medical image segmentation. Due to the complexity of medical images and the high requirement for annotation accuracy, the annotation task must be manually performed by experienced experts, which is not only time-consuming but also costly. Therefore, in medical image segmentation, the reality often involves having only a small amount of labeled data available, while there is a relatively large amount of unlabeled data. Many semi-supervised segmentation algorithms have limited ability to learn features from unlabeled data under the condition of a small amount of labeled data, leading to low accuracy in image segmentation.

To address the above issues, a semi-supervised medical image segmentation model based on deep consistency collaborative learning, named DCCLNet, is proposed. This model combines the consistency learning performance of feature perturbation and input perturbation and effectively utilizes the advantages of different paradigms learning in collaborative training. The consistency learning strategy is mainly reflected in the addition of different auxiliary decoders on the basis of the main network, enabling the auxiliary main network to learn. Meanwhile, the MT architecture, consisting of a teacher model and the main network, is used, with the teacher model guiding the main network’s learning, whereas the main network acts as the student model. The collaborative training framework consists of two different network architectures, CNN and ViT, which are trained simultaneously and learn from each other.

## Figures and Tables

**Table 1:** Different segmentation algorithms compare results on ACDC 5% and 10% labeled data.

Labeled Data	Method	RV			Myo			RV			Mean		
		DSC	HD_95_	ASD	DSC	HD_95_	ASD	DSC	HD_95_	ASD	DSC	HD_95_	ASD
5%	MT [11]	0.425	60.1	16.8	0.586	32.4	7.2	0.625	46.3	11.5	0.545	46.3	11.5
3/70	EM [39]	0.415	40.5	11.5	0.573	20.4	4.6	0.671	25.8	6.2	0.553	29.3	7.4
	DAN [24]	0.492	44.6	17.8	0.527	37.6	9.6	0.601	38.0	8.0	0.540	40.1	11.8
	UAMT [40]	0.417	44.0	14.2	0.557	29.5	6.8	0.613	31.1	7.3	0.529	34.9	9.4
	ICT [21]	0.436	29.2	11.2	0.573	21.6	5.5	0.623	25.2	7.2	0.544	25.3	8.0
	URPC [41]	0.387	38.9	15.5	0.441	25.7	7.2	0.545	32.9	11.7	0.458	32.5	11.5
	CPS [25]	0.425	33.8	8.5	0.569	20.2	4.6	0.653	23.1	3.5	0.549	25.7	6.2
	CCT [10]	0.467	34.4	12.0	0.539	18.9	4.7	0.639	21.1	5.8	0.548	24.8	7.5
	DCT [27]	0.374	40.3	12.7	0.494	22.5	6.0	0.553	25.3	7.4	0.473	20.4	**1.7**
	RD [23]	0.376	36.0	11.8	0.437	23.7	5.4	0.501	26.2	6.3	0.438	28.6	7.8
	CTCT [26]	0.677	17.6	5.1	0.642	12.6	3.0	0.750	14.1	3.4	0.690	14.7	3.9
	Ours	**0.734**	**14.8**	**3.5**	**0.738**	**15.6**	**3.0**	**0.832**	**9.3**	**2.1**	**0.768**	**13.2**	2.9
10%	MT	0.791	15.5	2.7	0.764	33.3	4.8	0.832	20.0	3.9	0.796	22.9	3.8
7/70	EM	0.743	**3.9**	1.1	0.798	7.8	1.4	0.849	11.0	2.0	0.797	7.6	1.5
	DAN	0.799	8.8	1.4	0.795	6.3	1.1	0.845	11.6	2.1	0.813	8.9	1.5
	UAMT	0.772	8.3	1.3	0.796	11.5	1.8	0.849	15.7	2.7	0.806	11.8	2.0
	ICT	0.815	5.1	1.1	0.809	10.7	1.6	0.850	16.5	2.8	0.825	10.8	1.8
	URPC	0.817	8.7	1.9	0.812	8.3	1,4	0.886	11.7	2.3	0.838	9.6	1.9
	CPS	0.831	**3.9**	**0.8**	0.826	6.6	1.3	0.871	13.1	2.3	0.843	7.9	1.5
	CCT	0.837	5.1	0.9	0.820	6.4	1.2	0.878	11.3	1.8	0.845	7.6	1.3
	DCT	0.757	5.9	1.3	0.762	36.1	5.8	0.855	17.8	2.6	0.792	19.9	3.2
	RD	0.814	6.6	1.3	0.810	7.4	1.2	0.869	11.0	2.0	0.831	8.4	1.5
	CTCT	0.861	5.0	1.1	0.841	6.3	1.0	0.895	13.5	1.8	0.866	8.3	1.3
	Ours	**0.888**	6.8	1.3	**0.861**	**4.8**	**1.0**	**0.921**	**6.5**	**1.4**	**0.890**	**6.0**	**1.2**

**Table 2:** Compare the results of different segmentation algorithms on Prostate 10% and 20% labeled data.

Labeled Data	Method	Mean		
		DSC	HD_95_	ASD
10% 4/35	MT	0.424	94.5	25.7
	EM	0.491	85.3	22.2
	DAN	0.568	96.6	23.5
	UAMT	0.490	91.2	26.7
	ICT	0.623	65.9	16.2
	URPC	0.317	65.1	24.5
	CPS	0.324	60.0	15.8
	CCT	0.409	57.3	21.4
	DCT	0.410	85.6	24.3
	RD	0.432	57.6	22.3
	CTCT	0.764	25.2	7.8
	Ours	**0.792**	**21.2**	**7.3**
20% 7/35	MT	0.635	35.1	11.6
	EM	0.620	41.6	13.2
	DAN	0.695	64.9	13.5
	UAMT	0.639	30.2	10.7
	ICT	0.734	26.5	9.2
	URPC	0.642	35.3	12.7
	CPS	0.602	47.1	13.6
	CCT	0.572	87.7	21.9
	DCT	0.659	36.1	12.1
	RD	0.633	39.6	12.8
	CTCT	0.783	26.9	8.4
	Ours	**0.812**	**19.3**	**6.4**

**Table 3:** Comparison of ACDC 10% labeled data network structure ablation results.

Method		RV			Myo			LV			Mean	
	DSC	HD_95_	ASD	DSC	HD_95_	ASD	DSC	HD_95_	ASD	DSC	HD_95_	ASD
UNet	0.673	16.3	3.6	0.785	10.0	1.7	0.874	13.8	2.6	0.778	13.8	2.6
UNet + Aux	0.791	15.5	2.7	0.764	33.3	4.8	0.832	20.0	3.9	0.796	22.9	3.8
UNet + Tea	0.837	5.1	1.9	0.820	6.4	1.2	0.878	11.3	1.8	0.845	7.6	1.3
UNet + ViT	0.861	5.0	1.1	0.841	6.3	**1.0**	0.895	13.5	1.8	0.866	8.3	1.3
UNet + Tea + Aux	0.801	10.1	2.0	0.808	10.2	1.7	0.871	21.3	3.2	0.826	13.9	2.3
UNet + Tea + ViT	0.870	7.4	1.5	0.857	7.1	1.2	0.913	11.7	2.0	0.880	8.7	1.6
UNet + Aux + ViT	0.880	**6.8**	1.4	0.860	**4.1**	**1.0**	0.910	9.4	1.6	0.884	6.8	1.3
DCCLNet	**0.888**	**6.8**	**1.3**	**0.861**	4.8	**1.0**	**0.921**	**6.5**	**1.4**	**0.890**	**6.0**	**1.2**

**Table 4:** Comparison of Prostate 20% labeled data network structure ablation results.

Method	Mean		
	DSC	HD_95_	ASD
UNet	0.563	95.3	24.6
UNet + Aux	0.572	87.7	21.9
UNet + Tea	0.635	35.1	11.6
UNet + ViT	0.783	26.9	8.4
UNet + Tea + Aux	0.695	33.2	10.1
UNet + Tea + ViT	0.794	24.6	7.9
UNet + Aux + ViT	0.807	21.0	7.2
DCCLNet	**0.812**	**19.3**	**6.4**

## Data Availability

The ACDC dataset and Prostate dataset are publicly available at https://github.com/HiLab-git/SSL4MIS (accessed on 10 October 2022).
